# Cumulus Expansion and Oocyte Quality Across the Pubertal Transition in Mice: Implications for Modeling Fertility Preservation in Adolescents

**DOI:** 10.21203/rs.3.rs-9658129/v1

**Published:** 2026-06-24

**Authors:** Dilan Gokyer, Luhan T. Zhou, Sophia Akinboro, Elnur Babayev

**Affiliations:** Northwestern University; Northwestern University; Northwestern University; Northwestern University

**Keywords:** Adolescent, pubertal transition, cumulus expansion, aneuploidy, oocyte competence

## Abstract

**Objective:**

To determine whether cumulus expansion and oocyte aneuploidy differ across the pubertal transition in a murine model designed to approximate clinical assisted reproductive technology (ART) conditions.

**Methods:**

Controlled experimental animal study using prepubertal (D16–25), peripubertal (D26–35), and reproductively young adult CD-1 female mice (9 and 12 weeks old). Mice underwent gonadotropin stimulation to model controlled ovarian hyperstimulation. Cumulus–oocyte complexes (COCs) were collected following in vivo maturation prior to ovulation or assessed before and after in vitro maturation (IVM). Main outcome measures included COC surface area, cumulus cell layer thickness, oocyte spindle configuration, chromosome alignment, and aneuploidy rates.

**Results:**

Following in vivo maturation, COC surface area (1505 ± 151.4 μm^2^ vs. 1735 ± 115.7 μm^2^ vs. 1637 ± 75.6 μm^2^, p = 0.44), cumulus layer thickness (176 ± 18 μm vs. 201.5 ± 21.2 μm vs. 196.7 ± 13.7 μm, p = 0.59), and oocyte euploidy rates (94.25 ± 3.6% vs. 91.41 ± 4.8% vs. 88.14 ± 2.9%, p = 0.57) were similar across D21–25, D28–35, and 12-week cohorts, respectively. Under IVM conditions, pre-IVM COC area was smaller in D16–21 mice compared with 9-week mice (377.6 ± 5.6 μm^2^ vs. 466.8 ± 16.5 μm^2^, p < 0.05). However, post-IVM COC area, cumulus expansion, and spindle/chromosome abnormality rates did not differ among groups (all p > 0.05).

**Conclusion:**

In this murine model, markers of oocyte competence, including cumulus expansion, meiotic integrity, and aneuploidy rates, were largely preserved across the pubertal transition, highlighting translational limitations of mice for modeling human adolescent ovarian biology.

## Introduction

Advances in cancer therapy and expanding fertility preservation strategies have led to a growing number of adolescents undergoing oocyte cryopreservation prior to gonadotoxic treatments [1, 2]. In addition, fertility preservation is offered to transgender and gender-diverse youth before initiation of pubertal suppression or gender-affirming hormone therapy [3]. While controlled ovarian hyperstimulation (COH) and oocyte vitrification are well established in reproductively adult women, substantially less is known about the biological competence of oocytes retrieved during early post-pubertal years. Adolescence represents a unique developmental window marked by endocrine maturation and establishment of ovulatory cyclicity [4], yet whether the ovary has achieved full functional competence immediately after pubertal transition remains incompletely understood.

Reproductive capacity follows a dynamic trajectory across the lifespan. While advanced reproductive age is well recognized to impair gamete competence through increased aneuploidy, mitochondrial dysfunction, and ovarian stromal inflammation [5–9], emerging evidence suggests that fertility may also be suboptimal at the earliest stages of reproductive life [10, 11]. Historical demographic analyses have documented lower pregnancy rates among very young females compared to reproductively adult women, including a large cohort from the Utah Population Database demonstrating an inverse U-shaped fertility curve [12–15]. Contemporary studies similarly suggest increased oocyte aneuploidy at the younger end of the reproductive spectrum, with higher chromosomal abnormality rates reported among women in their early 20s and younger [10, 16]. Similarly, studies in mammalian models indicate that juvenescent animals may exhibit altered reproductive outcomes and increased chromosomal abnormalities [17–21]. Together, these observations raise the possibility that full gamete competence may not be immediately established following pubertal transition.

While demographic data and oocyte and embryo analysis demonstrate lower gamete competence in very young humans, the biological mechanisms underlying these differences remain incompletely understood. The oocyte does not function in isolation; its developmental competence is tightly regulated by bidirectional communication with surrounding cumulus cells (CCs) and by the composition of follicular fluid (FF), which together constitute the immediate oocyte microenvironment [22]. In our recent study comparing adolescents undergoing fertility preservation with adult oocyte donors, we observed significant dysregulation in CC gene expression and a more pro-inflammatory cytokine milieu in FF of adolescents [23]. These findings demonstrated that the molecular profile of the oocyte microenvironment differs between early post-pubertal individuals and reproductively mature adults likely reflecting suboptimal quality of adolescent oocytes. However, direct functional assessment of human adolescent oocytes is not feasible, as these gametes are cryopreserved for future reproductive use. Therefore, experimental models are required to interrogate oocyte quality during this period.

Mouse provides a well-characterized model for studying ovarian physiology, with clearly defined pubertal timing and established protocols for controlled ovarian stimulation [24, 25]. Importantly, murine models allow direct assessment of key indicators of oocyte competence such as cumulus–oocyte complex (COC) expansion, meiotic spindle organization, chromosome alignment, and aneuploidy rates [26, 27]. Whether mouse can serve as a model to study adolescent oocyte quality in assisted reproductive technology (ART) cycles remains unclear.

In this study, we tested the hypothesis that the period of pubertal transition in mice can serve as a model to study human adolescent ovarian biology in ART cycles. Using a controlled experimental design, we modeled human COH in prepubertal, peripubertal, and reproductively young adult mice. We evaluated COC expansion in vivo and in vitro, assessed meiotic spindle morphology and chromosome alignment, and quantified aneuploidy rates in associated oocytes. Through this approach, we aimed to determine whether the pubertal transition influences markers of egg quality.

## Materials & Methods

### Animals

Reproductively young adult (9 and 12 weeks old), peripubertal (D26–35), and prepubertal (D16–25) CD-1 female mice were obtained from Envigo (Indianapolis, IN, USA). Mice were housed in a temperature-, humidity- and light- (14h light: 10h dark) controlled barrier facility within Northwestern University’s Center of Comparative Medicine. Upon arrival at Northwestern University, mice were fed with a diet formulated to exclude soybean meal, Teklad Global 2916 chow (Envigo, Madison, WI, USA), to avoid phytoestrogens. Mice were provided with food and water *ad libitum*. All animal experiments were approved by the Institutional Animal Care and Use Committee (IACUC) at Northwestern University (Protocol #IS00024694) and were conducted in accordance with institutional guidelines and the National Institutes of Health Guide for the Care and Use of Laboratory Animals.

#### COC Collection and Imaging following In Vivo Maturation

To model controlled ovarian hyperstimulation cycles utilized for human ART, mice were first hyperstimulated through intraperitoneal (IP) injections of 5 IU pregnant mare serum gonadotropin (PMSG; ProSpec-Tany TechnoGene, Cat. No. HOR-272). After 44–46 hours post-PMSG injection, mice underwent superovulation through IP injection of 5 IU human chorionic gonadotropin (hCG) (Sigma-Aldrich, St. Louis, MO, USA). After 10–11 hours post-hCG injection, ovaries were isolated and placed into dishes containing collection media consisting of Leibovitz’s L-15 medium (Life Technologies, Grand Island, NY, USA) supplemented with 3 mg/ml polyvinylpyrrolidone (PVP) (Sigma-Aldrich) and 0.5% penicillin-streptomycin (PS) (Life Technologies) (L15/PVP/PS). Antral follicles were mechanically punctured with insulin syringes to release COCs from the ovaries into the collection medium, to model oocyte retrieval after the trigger injection in human ART cycles. Transmitted light images of COCs were then captured for all age cohorts on an EVOS FL Auto Cell Imaging System (ThermoFisher Scientific, Waltham, MA, USA). These images were used to measure the COC area and cumulus cell layer thickness as described in **Supplementary Fig. 1**. COCs were transferred to a 1000 μL drop of L15/PVP/PS media containing 1 μM hyaluronidase (Sigma-Aldrich) to facilitate cumulus cell separation. Cumulus cells were denuded from the COCs through mechanical disruption to release the egg. Hyaluronidase was removed by rinsing the eggs through large drops of L15/PVP/PS media. Eggs were then used for downstream kinetochore analyses.

#### COC Collection and Imaging for In vitro Maturation (IVM)

Mice were hyperstimulated with IP injections of 5 IU PMSG. After 44–46 hours post-PMSG injection, ovaries were isolated and placed into dishes containing collection media consisting of L15/PVP/PS and 2.5 μM milrinone (Sigma-Aldrich), a phosphodiesterase 3A (PDE3A) inhibitor that maintains oocytes arrested at prophase of meiosis I [28]. Antral follicles were mechanically punctured with insulin syringes to release COCs from the ovaries into the collection media. Transmitted light images of COCs were then captured for each age cohort on EVOS FL Auto Cell Imaging System (ThermoFisher Scientific). These images were used to measure COC area as described in **Supplementary Fig. 1**. To induce synchronous meiotic maturation, milrinone was removed through washes in L15/PVP/PS. COCs were transferred to IVM media that supports cumulus expansion: α-MEM/5% (v/v) Fetal Bovine Serum (FBS)/10 ng/mL Epidermal Growth Factor (EGF)/20 mM HEPES/0.25mM pyruvate (Sigma-Aldrich and ThermoFisher Scientific). COCs were cultured individually in 150 μL of media in wells of ultra-low attachment 96 well plates (Corning Costar, Cat. No. CLS7007; Millipore Sigma). This experimental design allows us to track and assess the expansion of individual COCs. Cumulus expansion was assessed through measurements as described in **Supplementary Fig. 1**. After IVM, COCs were transferred to a drop of L15/PVP/PS media containing 1 μM hyaluronidase (Sigma-Aldrich) to facilitate cumulus cell separation. Cumulus cells were denuded from the COC through mechanical disruption to release the egg. Hyaluronidase was removed by rinsing eggs through large drops of L15/PVP/PS media. Eggs were then used for meiotic spindle and chromosome alignment analysis.

##### Oocyte aneuploidy assessment

Denuded eggs were transferred to treatment dishes containing 100 μM Monastrol (Tocris Bioscience, Cat. No. 1305, Avonmouth, Bristol, UK), a kinesin-5 inhibitor, in α-MEM Glutamax (MEM) (Life Technologies, Grand Island, NY, USA) supplemented with 3 mg/ml Bovine Serum Albumin (BSA) (Sigma-Aldrich) and 0.5% PS (MEM/BSA/PS). Eggs were treated for 3 hours in a humified atmosphere of 5% CO_2_ in air at 37°C. This treatment leads to the collapse of bipolar spindles and allows the dispersion of chromosomes to facilitate chromosome and kinetochore number assessment [29]. Eggs were fixed, permeabilized, and blocked as previously described [30]. They were then incubated in a 1:100 dilution of human ANTI-Centromere (Kinetochore) antibody (Antibodies Incorporated, Cat. No. 15–234, Davis, CA, USA) overnight at 4°C followed by washes in blocking buffer (BB) containing PBS, 0.3% BSA, 0.01% Tween-20, and 0.02% sodium azide (NaN_3_). Alexa-Fluor 488-conjugated goat anti-human secondary antibody (Invitrogen, Carlsbad, CA, USA) was used for fluorescent detection. Oocytes were then washed in blocking solution and then mounted in Vectashield containing 4’,6-diamidino-2phenylindole (DAPI; Vector Laboratories, Burlingame, CA, USA) for chromosome staining. Eggs were imaged on a Leica SP5 inverted laser scanning confocal microscope (Leica Microsystems) using a 63X objective and the 405 and 488 nm lasers. For each egg, 0.5 μm optically thick sections were taken through the region of DNA and images were analyzed using ImageJ software (National Institutes of Health, Bethesda, MD, USA). Eggs containing 40 kinetochores were considered euploid and any deviation from this was considered aneuploid.

##### Evaluation of meiotic spindle morphology and chromosome alignment

Eggs were fixed in 3.8% paraformaldehyde (Electron Microscopy Science, Hatfield, PA, USA) in phosphate buffered saline (PBS) containing 0.1% Triton-X-100 for 1 hour at 37°C. Cells were then washed in BB. Following this, oocytes were permeabilized in PBS containing 0.3% BSA, 0.1% Triton-X-100, and 0.02% NaN_3_ for 15 minutes at room temperature, and then washed twice in BB. Oocytes were then probed overnight with a 1:100 dilution of mouse anti-pericentrin primary antibody (BD Biosciences, Cat. No. 611814, Franklin Lakes, NJ, USA). Pericentrin is a protein that localizes to microtubule-organizing centers and marks the spindle poles, enabling accurate visualization of spindle orientation [31] and ensuring that only spindles aligned within the imaging plane were included for chromosome alignment analysis. Oocytes were then washed three times in BB and microtubules were stained with a 1:100 dilution of Alexa Fluor 488-conjugated alpha-Tubulin rabbit monoclonal antibody (11H10; Cell Signaling Technology, Danvers, MA, USA) along with a 1:200 dilution of Alexa-Fluor 568 goat anti-mouse antibody (Invitrogen, Cat. No. A11004) for 2 hours at room temperature to visualize the pericentrin. Then cells were washed with BB and mounted onto glass slides in Vectashield containing DAPI. Eggs were imaged on a Leica SP5 confocal microscope as described above. ImageJ software was used for analysis (National Institutes of Health). Spindles that were oriented perpendicular to the image plane, rendering spindle assessment infeasible were excluded from the analysis. Similarly, only the eggs with spindles parallel to the imaging plane were used for this analysis to accurately determine chromosome alignment. Chromosome configuration was analyzed and categorized as follows: normal (no misaligned chromosomes), or abnormal with 1 or more misaligned chromosomes on the metaphase plate. The abnormal category also included the oocytes with abnormally shaped spindles (e.g., multipolar).

### Statistical Analysis

The normal distribution of the data was evaluated with the Shapiro-Wilk test. Analysis between the three groups of continuous variables was performed with ordinary one-way ANOVA or Kruskal-Wallis test depending on the distribution. Tukey’s or Dunn’s multiple comparisons test was used for post-hoc analysis. Data are presented as mean ± standard error of the mean (SEM). P values < 0.05 were considered statistically significant. GraphPad Prism version 9.0.1 (Boston, Massachusetts USA, www.graphpad.com) was used for statistical analysis.

## Results

### COCs expansion in vivo following hyperstimulation does not change across the pubertal transition in mice

**Figure 1:**
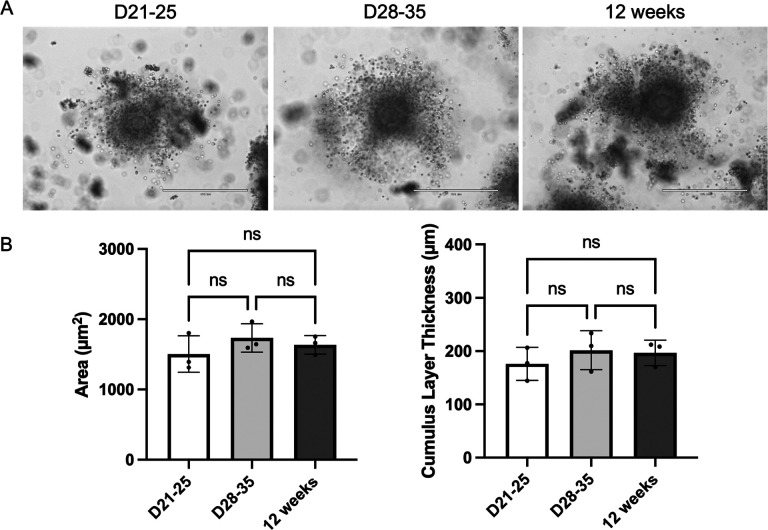
Expanded COCs following *in vivo* maturation do not significantly differ across the pubertal transition in mice. (A) Representative images of expanded COCs following *in vivo* maturation for each age cohort. Scale bars = 400μm. (B) Cumulus area and cumulus layer thickness do not significantly differ between age cohorts after *in vivo* maturation. n=3 replicates; averages of 8–15 COCs per replicate per age cohort. Data are presented as mean ± standard error of the mean (SEM).

To determine whether cumulus expansion varies across the pubertal transition, we analyzed in vivo matured COCs collected 10–11 hours after hCG administration from prepubertal (D21–25), peripubertal (D28–35), and reproductively young (12 weeks) CD-1 mice. This stimulation paradigm models controlled ovarian hyperstimulation and oocyte retrieval in human ART cycles. Representative images of expanded COCs in each age group are shown in Fig. 1A. Quantitative analysis revealed no significant differences in COC surface area among age cohorts. Mean COC area was 1505 ± 151.4 μm^2^ in D21–25 mice, 1735 ± 115.7 μm^2^ in D28–35 mice, and 1637 ± 75.6 μm^2^ in adult mice (p = 0.44). Similarly, average cumulus cell layer thickness did not differ across groups (176 ± 18 μm vs 201.5 ± 21.2 μm vs 196.7 ± 13.7 μm; p = 0.59) (Fig. 1B).

#### Oocyte aneuploidy rates following hyperstimulation do not significantly differ across the pubertal transition in mice

**Figure 2:**
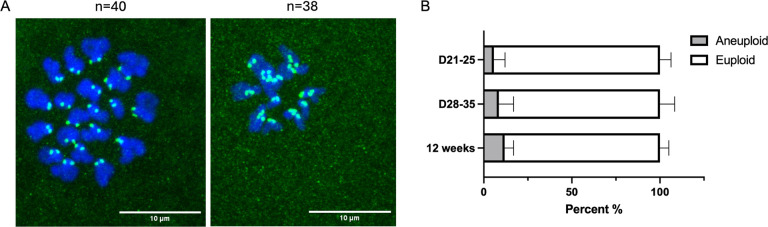
Euploidy rates in mature oocytes of in vivo expanded COCs are not significantly different across the pubertal transition in mice. (A) Representative images of a euploid (left) and aneuploid (right) kinetochore spreads; centromeres (green) and DNA (blue). Scale bar 10 μm. (B) Euploidy rates are not significantly different across the age cohorts. n=3 replicates; between 6–25 eggs per replicate. Data are presented as mean ± standard error of the mean (SEM).

Oocyte aneuploidy was assessed by kinetochore counting following in vivo maturation using the ART paradigm above (Fig. 2A). Euploidy did not differ significantly among prepubertal (D21–25), peripubertal (D28–35), and reproductively adult (12 weeks) mice oocytes. Mean euploidy rates were 94.25 ± 3.6%, 91.41 ± 4.8%, and 88.14 ± 2.9% for D21–25, D28–35, and adult mice, respectively (p = 0.57) (Fig. 2B).

#### COC expansion in vitro is similar across age groups during the pubertal transition in mice

**Figure 3:**
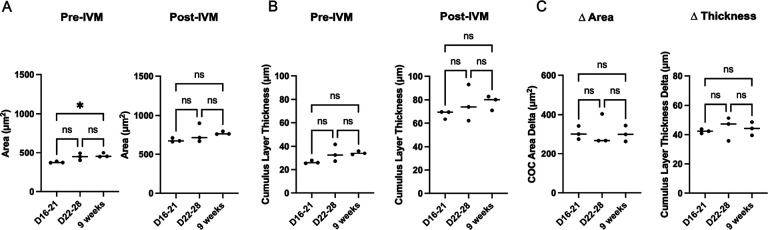
COC expansion is not significantly different during *in vitro* maturation (IVM) across the pubertal transition in mice. (A) COC area measurements prior to (left) and post IVM (right). Asterisk indicates a significant difference (p<0.05). (B) Cumulus layer thickness measurements prior to (left) and post IVM (right). (C) The delta between pre- and post-IVM measurements for COC area (left) and cumulus layer thickness (right). n=3 replicates; between 13–20 individually matured COCs per age cohort. Averages of replicates are shown on the graphs.

To assess whether cumulus expansion differs across pubertal transition under controlled in vitro conditions, COCs from prepubertal (D16–21 and D22–28) and reproductively young adult (9 weeks) CD-1 mice were imaged prior to initiation of IVM and again following IVM (Fig. 3). Pre-IVM COC area differed by age. Mean COC area prior to IVM was 377.6 ± 5.6 μm^2^ in D16–21 mice, 448.6 ± 26.5 μm^2^ in D22–28 mice, and 466.8 ± 16.5 μm^2^ in 9-week mice, with the youngest cohort exhibiting a significantly smaller COC area compared to 9-week mice (p < 0.05). However, COC expansion was not different across groups. Post-IVM COC area (683.5 ± 14.7 μm^2^, 761.7 ± 69.8 μm^2^, and 773.4 ± 11.3 μm^2^ for D16–21, D22–28, and 9-week mice, respectively, p = 0.32) and ΔCOC (post-expansion area – pre-expansion area, 305.8 ± 19.4 vs. 313 ± 45.6 vs. 302.3 ± 23.2, p = 0.93) did not significantly differ across groups (Fig. 3A,C).

Average cumulus cell layer thickness was not different among age cohorts either prior to (26.4 ± 0.8 μm, 33.7 ± 4.2 μm, and 34.3 ± 0.9 μm, p = 0.12) or following IVM (67.6 ± 2.1 μm, 76.4 ± 9.0 μm, and 78.0 ± 3.5 μm, p = 0.3) (Fig. 3B). Similarly, the change in cumulus layer thickness (Δ thickness) did not differ among groups (42.35 ± 0.8 vs. 44.8 ± 4.6 vs. 44.1 ± 2.6, p = 0.85). (Fig. 3C). These data indicate that despite beginning with a smaller baseline area, prepubertal COCs retain intact cumulus expansion capacity during IVM.

##### Spindle configuration and chromosome alignment of in vitro matured eggs was not significantly different across the pubertal transition in mice

**Figure 4:**
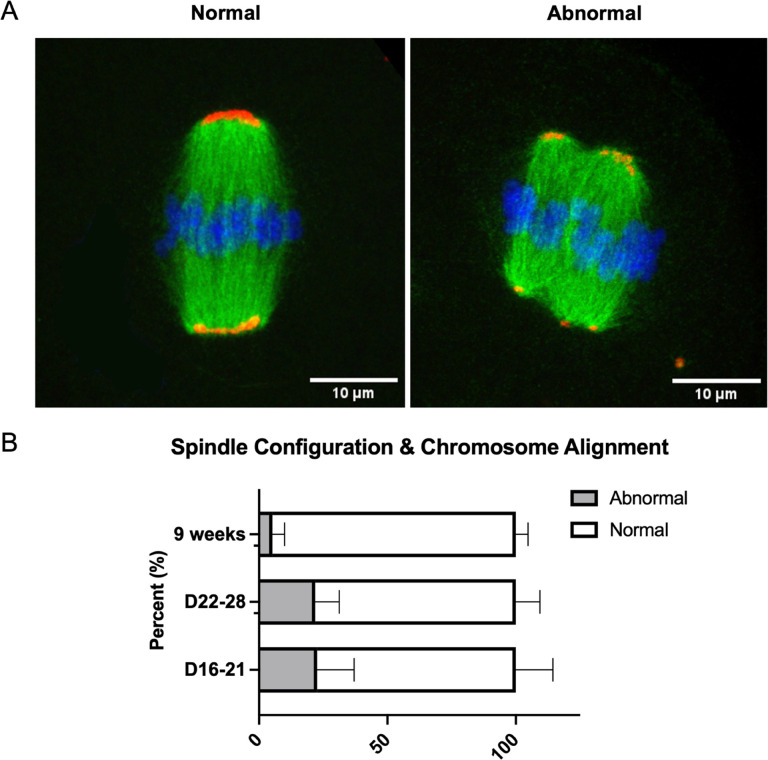
Spindle configuration and chromosome alignment in mature oocytes of in vitro expanded COCs are not significantly different across the pubertal transition in mice. (A) Representative images of a normal (left) and abnormal (right) spindle; tubulin (green), pericentrin (red), and DNA (blue). Scale bar 10μm. (B) Percent of mature oocytes that are either normal (both spindle morphology and chromosome alignment) or abnormal (spindle morphology and/or chromosomal alignment is abnormal). n=3 replicates; between 9–15 mature oocytes per replicate per age cohort. Data are presented as mean ± standard error of the mean (SEM).

Spindle morphology and chromosome alignment were evaluated as markers of meiotic integrity and oocyte competence in in vitro matured oocytes from D16–21, D22–28, and 9-week CD-1 mice (Fig. 4A). The percentage of oocytes exhibiting abnormal spindle morphology and/or chromosome misalignment did not significantly differ across age cohorts. Mean abnormality rates were 22.6 ± 8.3% in D16–21 mice, 21.9 ± 5.4% in D22–28 mice, and 5.3 ± 2.7% in 9-week mice (p = 0.11) (Fig. 4B). A trend toward higher abnormality rates in in vitro–matured oocytes from juvenescent mice compared to adults is observed, contrasting with the euploidy rates seen in in vivo–matured oocytes. This discrepancy likely reflects fundamental differences between in vitro and in vivo maturation environments, with the latter more closely modeling physiologic oocyte development and clinical ART conditions in the mouse.

## Discussion

In this study, we investigated whether mouse can serve as a model to study human adolescent oocyte biology in fertility preservation cycles using experimental paradigms designed to approximate clinical ART. Across age cohorts spanning prepubertal/peripubertal to reproductively young adulthood, we observed no significant differences in COC expansion following in vivo maturation after controlled ovarian hyperstimulation. Kinetochore-based assessment likewise revealed no age-dependent differences in chromosomal segregation outcomes following in vivo maturation. Under controlled in vitro maturation (IVM) conditions, COCs from the youngest cohort exhibited a smaller baseline area prior to maturation. However, post-IVM COC area, cumulus layer thickness, and the magnitude of expansion were comparable across age groups. Finally, spindle configuration and chromosome alignment, markers of meiotic integrity and oocyte competence [5, 6, 10], did not differ across cohorts. Together, these data suggest that, within the mouse, key morphological and cytogenetic indicators of oocyte competence are largely preserved across the pubertal transition under gonadotropin-stimulated conditions.

These findings are relevant in the context of expanding fertility preservation practices in adolescents [3, 32–34]. In humans, direct functional assessment of adolescent oocytes is not feasible because retrieved oocytes are cryopreserved for future reproductive use. Consequently, long-term outcomes such as fertilization rates, embryo development, implantation, and live birth following use of adolescent-frozen oocytes remain limited. Experimental models therefore remain essential for interrogating whether pubertal maturation itself influences oocyte quality and meiotic integrity.

We initially employed an hCG-triggered in vivo maturation paradigm to closely approximate the clinical timing of final oocyte maturation and retrieval during human ART cycles, in which oocytes are collected following a trigger injection and prior to ovulation [35]. When this approach revealed no age-dependent differences, we incorporated a controlled IVM paradigm to increase experimental precision. IVM allows longitudinal assessment of individual COCs and eliminates potential confounding from follicular wall components that may variably contribute to apparent COC boundaries following mechanical follicle puncture [36, 37]. We also refined age windows to better capture earlier developmental stages surrounding puberty, consistent with established frameworks describing murine pubertal timing [21]. Across both paradigms, however, functional readouts of oocyte competence remained comparable among age cohorts.

Notably, these murine findings contrast with molecular alterations we previously observed in the human adolescent oocyte microenvironment [23]. In that study, cumulus cells from adolescents undergoing fertility preservation demonstrated dysregulated transcriptomic signatures relative to adult oocyte donors, and follicular fluid exhibited a more pro-inflammatory cytokine profile [23]. Such microenvironmental differences could plausibly reflect variation in gamete competence at early reproductive ages in humans. The absence of parallel functional differences in the mouse suggests that species-specific biology may underlie this discrepancy arguing against the use of mice as a model to study oocyte quality in pediatric population.

A key translational consideration is that mice are polyovulatory and exhibit a compressed reproductive developmental timeline relative to humans [38]. Pubertal maturation and establishment of cyclicity occur over a brief interval, and the endocrine milieu of murine juvensence may not replicate the prolonged maturation observed in humans. Baseline aneuploidy rates are also generally low in young mice compared with humans [39], and mechanisms governing meiotic fidelity may differ across species [40, 41]. In addition, exogenous gonadotropin stimulation and IVM conditions may attenuate subtle developmental differences that could otherwise emerge under natural cycling conditions or through downstream developmental endpoints. Collectively, these factors suggest that while the mouse remains a powerful mechanistic model for studying meiotic regulation [26, 42, 43] and ovarian physiology [7, 8,44], it may not fully capture aspects of human adolescent ovarian biology relevant to fertility preservation.

A non-human primate model may offer greater translational relevance. Reproductive aging patterns in chimpanzees have been reported to more closely resemble those observed in humans, including age-related changes in fertility trajectories [15]. Such models may therefore better recapitulate human reproductive developmental dynamics. However, ethical, logistical, and financial constraints substantially limit the feasibility of large-scale experimental work in non-human primates.

This study has limitations. Our primary outcomes focused on morphological and cytogenetic markers of competence, and we did not evaluate downstream endpoints such as fertilization capacity, embryo development, or implantation. Furthermore, stimulation paradigms may obscure age-related differences that could be more evident under natural endocrine conditions.

In summary, across multiple experimental paradigms modeling ART-associated oocyte maturation, we did not detect meaningful age-dependent differences in COC expansion, spindle organization, chromosome alignment, or aneuploidy across the pubertal transition in mice. These findings highlight an important translational limitation in using the mouse as a model for human adolescent ovarian biology. Continued investigation of complementary models will be necessary to better define the reproductive potential of oocytes retrieved during early post-pubertal years in humans.

## Supplementary Material

Supplementary Files

This is a list of supplementary files associated with this preprint. Click to download.

- SupplementaryFig.1050726.pdf

## References

[1] Armstrong GT, Chen Y, Yasui Y, Leisenring W, Gibson TM, Mertens AC, Reduction in Late Mortality among 5-Year Survivors of Childhood Cancer. N Engl J Med. 2016;374:833–42. 10.1056/NEJMoa1510795. 20160113th ed.. 26761625 PMC4786452

[2] Goldman KN, Chenette D, Arju R, Duncan FE, Keefe DL, Grifo JA, mTORC1/2 inhibition preserves ovarian function and fertility during genotoxic chemotherapy. Proc Natl Acad Sci U S A. 2017;114:3186–91. 10.1073/pnas.1617233114. 20170307th ed.. 28270607 PMC5373380

[3] Mulder RL, Font-Gonzalez A, Hudson MM, van Santen HM, Loeffen EAH, Burns KC, Fertility preservation for female patients with childhood, adolescent, and young adult cancer: recommendations from the PanCareLIFE Consortium and the International Late Effects of Childhood Cancer Guideline Harmonization Group. Lancet Oncol. 2021;22:e45–56. 10.1016/S1470-2045(20)30594-5. 33539753

[4] WHO. World Health Organization: Adolescent health overview. 2024.

[5] Wang Z-B, Schatten H, Sun Q-Y. Why is chromosome segregation error in oocytes increased with maternal aging? Physiol (Bethesda). 2011;26:314–25. 10.1152/physiol.00020.2011. 22013190

[6] Duncan FE, Hornick JE, Lampson MA, Schultz RM, Shea LD, Woodruff TK. Chromosome cohesion decreases in human eggs with advanced maternal age. Aging Cell. 2012;11:1121–4. 10.1111/j.1474-9726.2012.00866.x. 22823533 PMC3491123

[7] Briley SM, Jasti S, McCracken JM, Hornick JE, Fegley B, Pritchard MT, Reproductive age-associated fibrosis in the stroma of the mammalian ovary. Reproduction. 2016;152:245–60. 10.1530/REP-16-0129. 27491879 PMC4979755

[8] Amargant F, Manuel SL, Tu Q, Parkes WS, Rivas F, Zhou LT, Ovarian stiffness increases with age in the mammalian ovary and depends on collagen and hyaluronan matrices. Aging Cell. 2020;19:e13259. 10.1111/acel.13259. 33079460 PMC7681059

[9] Beverley R, Snook ML, Brieño-Enríquez MA. Meiotic Cohesin and Variants Associated With Human Reproductive Aging and Disease. Front Cell Dev Biol. 2021;9:710033. 10.3389/fcell.2021.710033. 34409039 PMC8365356

[10] Gruhn JR, Zielinska AP, Shukla V, Blanshard R, Capalbo A, Cimadomo D Chromosome errors in human eggs shape natural fertility over reproductive life span. Science (1979). 2019;365:1466–9. 10.1126/science.aav7321 PMC7212007 31604276

[11] Duncan FE. Egg Quality during the Pubertal Transition-Is Youth All It’s Cracked Up to Be? Front Endocrinol (Lausanne). 20170904th ed. 2017;8:226. 10.3389/fendo.2017.00226 28928717 PMC5591325

[12] Hartman CG. On the Relative Sterility of the Adolescent Organism. Science (1979). 1931;74:226–7. 10.1126/science.74.1913.226 17834965

[13] Ashley-Montagu MF, Adolescent Sterility. Q Rev Biol. Volume 14. University of Chicago Press; 1939. pp. 13–34.

[14] Wood JW, Milligan SM. Oxford reviews of reproductive biology. New York: Oxford University Press; 1989.

[15] Hawkes K, Smith KR. Do women stop early? Similarities in fertility decline in humans and chimpanzees. Ann N Y Acad Sci. 2010;1204:43–53. 10.1111/j.1749-6632.2010.05527.x. 20738274 PMC4043631

[16] Franasiak JM, Forman EJ, Hong KH, Werner MD, Upham KM, Treff NR The nature of aneuploidy with increasing age of the female partner: a review of 15,169 consecutive trophectoderm biopsies evaluated with comprehensive chromosomal screening. Fertil Steril. 20131217th ed. 2014;101:656–663 e1. 10.1016/j.fertnstert.2013.11.004 24355045

[17] Mirskaia L, Crew FAE. XIV.—Maturity in the Female Mouse. Proceedings of the Royal Society of Edinburgh. 2014/09/15. Royal Society of Edinburgh Scotland Foundation; 1931;50:179–86. 10.1017/S0370164600044850

[18] Koenig JL, Stormshak F. Cytogenetic evaluation of ova from pubertal and third-estrous gilts. Biol Reprod. 1993;49:1158–62. 10.1095/biolreprod49.6.1158. 8286598

[19] Wallen K, Zehr JL. Hormones and history: the evolution and development of primate female sexuality. J Sex Res. 2004;41:101–12. 10.1080/00224490409552218. 15216429 PMC1255935

[20] Lechniak D, Warzych E, Pers-Kamczyc E, Sosnowski J, Antosik P, Rubes J. Gilts and sows produce similar rate of diploid oocytes in vitro whereas the incidence of aneuploidy differs significantly. Theriogenology. 2007;68:755–62. 10.1016/j.theriogenology.2007.06.012. 20070712th ed.. 17628654

[21] Kusuhara A, Babayev E, Zhou LT, Singh VP, Gerton JL, Duncan FE. Immature Follicular Origins and Disrupted Oocyte Growth Pathways Contribute to Decreased Gamete Quality During Reproductive Juvenescence in Mice. Front Cell Dev Biol. 2021;9:693742. 10.3389/fcell.2021.693742. 20210616th ed.. 34222262 PMC8244820

[22] Babayev E, Duncan FE. Age-associated changes in cumulus cells and follicular fluid: the local oocyte microenvironment as a determinant of gamete quality. Biol Reprod. 2022;106:351–65. 10.1093/biolre/ioab241. 34982142 PMC8862720

[23] Gokyer D, Akinboro S, Zhou LT, Kleinhans A, Laronda MM, Duncan FE, The oocyte microenvironment is altered in adolescents compared to oocyte donors. Hum Reprod Open. 2024;2024:hoae047. 10.1093/hropen/hoae047. 39211054 PMC11361810

[24] Gaytan F, Morales C, Leon S, Heras V, Barroso A, Avendaño MS, Development and validation of a method for precise dating of female puberty in laboratory rodents: The puberty ovarian maturation score (Pub-Score). Sci Rep. 2017;7:46381. 10.1038/srep46381. 28401948 PMC5388887

[25] Luo C, Zuñiga J, Edison E, Palla S, Dong W, Parker-Thornburg J. Superovulation strategies for 6 commonly used mouse strains. J Am Assoc Lab Anim Sci. 2011;50:471–8. 21838974 PMC3148645

[26] Suebthawinkul C, Babayev E, Zhou LT, Lee HC, Duncan FE. Quantitative morphokinetic parameters identify novel dynamics of oocyte meiotic maturation and cumulus expansion†. Biol Reprod. 2022;107:1097–112. 10.1093/biolre/ioac139. 35810327 PMC9562117

[27] Pan H, Ma P, Zhu W, Schultz RM. Age-associated increase in aneuploidy and changes in gene expression in mouse eggs. Dev Biol. 2008;316:397–407. 10.1016/j.ydbio.2008.01.048. 18342300 PMC2374949

[28] Gilchrist RB, Luciano AM, Richani D, Zeng HT, Wang X, Vos M, De, Oocyte maturation and quality: role of cyclic nucleotides. Reproduction. 2016;152:R143–57. 10.1530/REP-15-0606. 27422885

[29] Mayer TU, Kapoor TM, Haggarty SJ, King RW, Schreiber SL, Mitchison TJ. Small molecule inhibitor of mitotic spindle bipolarity identified in a phenotype-based screen. Science. 1999;286:971–4. 10.1126/science.286.5441.971. 10542155

[30] Duncan FE, Chiang T, Schultz RM, Lampson MA. Evidence that a defective spindle assembly checkpoint is not the primary cause of maternal age-associated aneuploidy in mouse eggs. Biol Reprod. 2009;81:768–76. 10.1095/biolreprod.109.077909. 19553597 PMC2754889

[31] Ma W, Viveiros MM. Depletion of pericentrin in mouse oocytes disrupts microtubule organizing center function and meiotic spindle organization. Mol Reprod Dev. 2014;81:1019–29. 10.1002/mrd.22422. 25266793 PMC4229429

[32] Michalczyk K, Cymbaluk-Płoska A. Fertility Preservation and Long-Term Monitoring of Gonadotoxicity in Girls, Adolescents and Young Adults Undergoing Cancer Treatment. Cancers (Basel). 2021;13. 10.3390/cancers13020202. PMC7827074 33429908

[33] Chen D, Simons L. Ethical considerations in fertility preservation for transgender youth: A case illustration. Clin Pract Pediatr Psychol. 2018;6:93–100. 10.1037/cpp0000230. 29963344 PMC6023412

[34] ASRM Committee Opinion. Fertility preservation in patients undergoing gonadotoxic therapy or gonadectomy: a committee opinion. Fertil Steril. 2019;112:1022–33. 10.1016/j.fertnstert.2019.09.013. 31843073

[35] ASRM Committee Opinion. Evidence-based outcomes after oocyte cryopreservation for donor oocyte in vitro fertilization and planned oocyte cryopreservation: a guideline. Fertil Steril. 2021;116:36–47. 10.1016/j.fertnstert.2021.02.024. 34148587

[36] Salustri A, Yanagishita M, Underhill CB, Laurent TC, Hascall VC. Localization and synthesis of hyaluronic acid in the cumulus cells and mural granulosa cells of the preovulatory follicle. Dev Biol. 1992;151:541–51. 10.1016/0012-1606(92)90192-J. 1601185

[37] Turathum B, Gao E-M, Chian R-C. The Function of Cumulus Cells in Oocyte Growth and Maturation and in Subsequent Ovulation and Fertilization. Cells. 2021;10. 10.3390/cells10092292. PMC8470117 34571941

[38] Caligioni CS. Assessing reproductive status/stages in mice. Curr Protoc Neurosci. 2009. 10.1002/0471142301.nsa04is48. Appendix 4:Appendix 4I. PMC2755182 19575469

[39] Camlin NJ, McLaughlin EA, Holt JE. The use of C57Bl/6 × CBA F1 hybrid cross as a model for human age-related oocyte aneuploidy. Mol Reprod Dev. 2017;84:6–7. 10.1002/mrd.22766. 27935143

[40] Huang W, Li X, Yang H, Huang H. The impact of maternal age on aneuploidy in oocytes: Reproductive consequences, molecular mechanisms, and future directions. Ageing Res Rev. 2024;97:102292. 10.1016/j.arr.2024.102292. 38582380

[41] Zhu Y, Kratka CR, Pea J, Lee HC, Kratka CE, Xu J, The severity of meiotic aneuploidy is associated with altered morphokinetic variables of mouse oocyte maturation. Hum Reprod Open. 2024;2024:hoae023. 10.1093/hropen/hoae023. 38764910 PMC11099657

[42] Zhou LT, Romar R, Pavone ME, Soriano-Úbeda C, Zhang J, Slawson C, Disruption of O-GlcNAc homeostasis during mammalian oocyte meiotic maturation impacts fertilization. Mol Reprod Dev. 2019;86:543–57. 10.1002/mrd.23131. 30793403 PMC6510634

[43] Valsangkar D, Downs SM. A requirement for fatty acid oxidation in the hormone-induced meiotic maturation of mouse oocytes. Biol Reprod. 2013;89:43. 10.1095/biolreprod.113.109058. 23863407 PMC4076365

[44] Amargant F, Zhou LT, Yuan Y, Nahar A, Krisher RL, Spate LD, FGF2, LIF, and IGF1 (FLI) supplementation during human in vitro maturation enhances markers of gamete competence. Hum Reprod. 2023;38:1938–51. 10.1093/humrep/dead162. 37608600

